# Personalized Nanomedicine: A Revolution at the Nanoscale

**DOI:** 10.3390/jpm7040012

**Published:** 2017-10-12

**Authors:** Cristina Fornaguera, Maria José García-Celma

**Affiliations:** 1 Sagetis Biotech, 08017 Barcelona, Spain; 2 Department of Pharmacy and Pharmaceutic Technology and Physicochemistry, R + D Associated Unit to CSIC, Faculty of Pharmacy and Food Sciences and Institute of Nanosciences and Nanotechnology (IN2UB), University of Barcelona, 08028 Barcelona, Spain; Centro de Investigación Biomédica en Red (CIBER) of Bioengineering, Biomaterials and Nanomedicine (CIBER-BBN), 08028 Barcelona, Spain

**Keywords:** personalized nanomedicine, nanotherapeutics, nanodrugs, nanosystems, scale-up, nanomedicines market, stakeholders

## Abstract

Nanomedicine is an interdisciplinary research field that results from the application of nanotechnology to medicine and has the potential to significantly improve some current treatments. Specifically, in the field of personalized medicine, it is expected to have a great impact in the near future due to its multiple advantages, namely its versatility to adapt a drug to a cohort of patients. In the present review, the properties and requirements of pharmaceutical dosage forms at the nanoscale, so-called nanomedicines, are been highlighted. An overview of the main current nanomedicines in pre-clinical and clinical development is presented, detailing the challenges to the personalization of these therapies. Next, the process of development of novel nanomedicines is described, from their design in research labs to their arrival on the market, including considerations for the design of nanomedicines adapted to the requirements of the market to achieve safe, effective, and quality products. Finally, attention is given to the point of view of the pharmaceutical industry, including regulation issues applied to the specific case of personalized medicine. The authors expect this review to be a useful overview of the current state of the art of nanomedicine research and industrial production, and the future opportunities of personalized medicine in the upcoming years. The authors encourage the development and marketing of novel personalized nanomedicines.

## 1. Introduction

**Figure 1:**
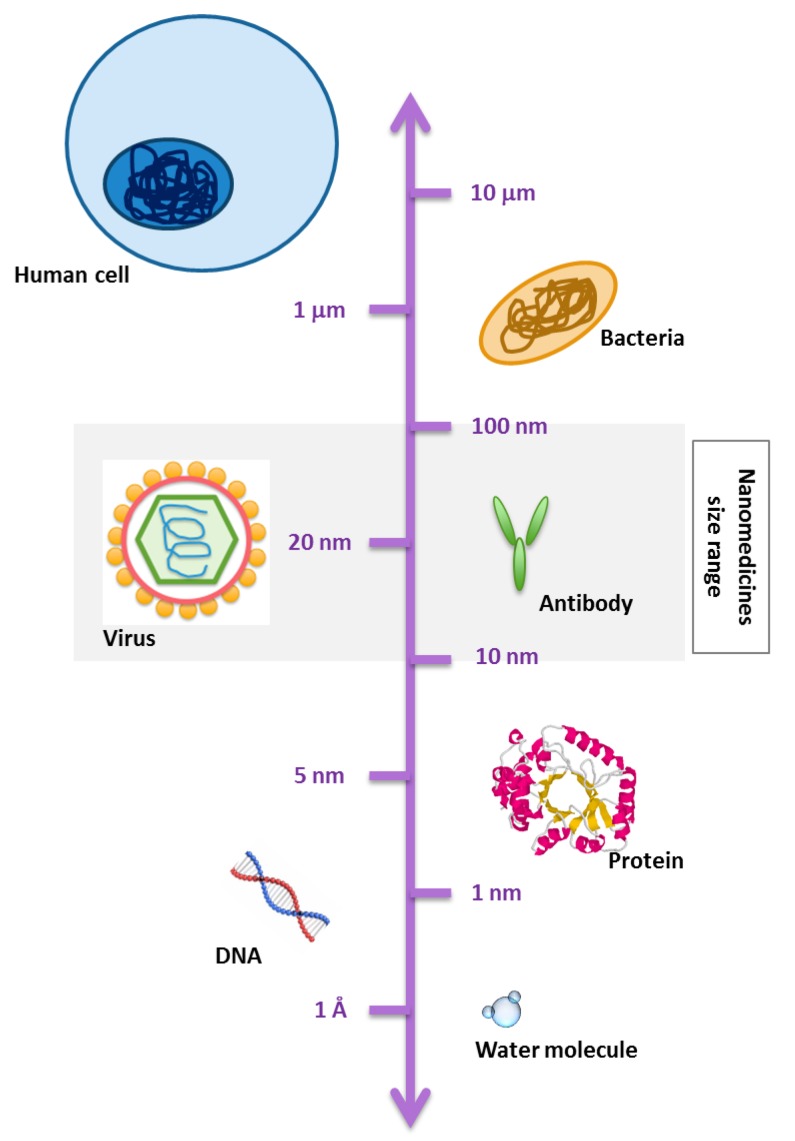
Schematic representation of a scale bar to visualize the range of nanomaterials and nanosystems as compared with biological components.

Nanomedicines are nowadays defined as nanoscale tools (e.g., 1–1000 nm sized) for the diagnosis, prevention, and treatment of diseases [1,2,3], having the potential to enable early detection and prevention of diseases, guide a bioactive molecule to its desired location of action, and/or control its release to ensure an optimal concentration at the therapeutic target over a desired time frame [2,4,5,6]. In this context, nanomedicines can be included in the field of “precision medicine,” which consists of a very sensitive diagnosis and targeted treatment of diseases [3]. Nanomedicines are also reported as nanodrugs, nanocarriers, nanoconstructs, nanoparticles, nanomaterials, or nanotherapeutics [7,8,9,10,11,12,13]. In general, the terms “nanomaterial” and “nanosystem” include a wide variety of objects with at least one dimension in the nanometric scale (typically 1–1000 nm) (Figure 1). The term nanomedicine appeared in the 1990s, and since then, its interest has experienced an exponential increase within the scientific community, notably during the last 20 years, after the government of the United States announced that it was starting to fund nanomedicinal studies [7,8,14,15,16,17,18,19,20,21]. More recently, the European Technology platform on Nanomedicine (ETPN) defined the term “nanomedicine” as: “The application of nanotechnology to achieve breakthroughs in healthcare that exploits the improved and often novel physical, chemical and biological properties of materials at the nanometer scale” [4]. The European Medicines Agency (EMA), the EU agency responsible for the scientific evaluation and supervision of medicines, refers to nanomedicine as “a system designed for clinical applications that is composed of at least one nanoscale component, resulting in specific advantageous characteristics, such as better targeting and bioavailability of therapeutics, new modes of therapeutic action, and nanostructured surfaces/scaffolds for engineered tissues” [10,22].

However, the central idea of nanomedicine—to produce a therapeutic effect specifically in the target organ—is not new. Almost a century ago, Paul Ehrlich introduced the concept of “magic bullet” [3]: an entity able to recognize a target and provide a therapeutic action in this specific target [4,23,24]. A particular tissue, cell type, or disease marker is the main target in precision medicine [3]. Currently, the concept of magic bullet includes a coordinated behavior of three components: (a) drug; (b) targeting moiety; and (c) pharmaceutical carrier used to multiply the number of drug molecules per single targeting moiety. Following this idea, research studies concerning nanomedicine applications in the biomedical field are numerous, from their use as therapeutic agents to treat any imaginable disease to their use as diagnostic systems [4,15,25,26,27,28,29]. A special field is the use of nanomedicines, due to their tunable physicochemical properties, for the design of novel personalized therapies [4].

**Figure 2:**
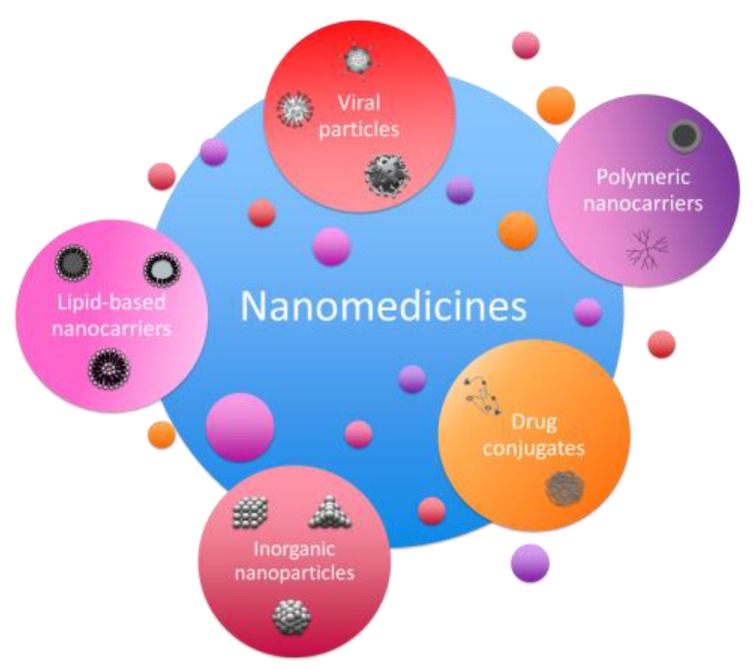
Schematic representation of different types of nanomedicines.

Personalized medicine can be defined as a healthcare strategy that aims at the development of specific treatments for each patient/group of patients, taking into account genetic, phenotypic, and environmental factors that could influence the outcome (efficacy and safety) of the therapy [4]. The use of nanomedicines in this field has also experienced an exponential increase since it represents an opportunity to treat each individual or each group of individuals with common characteristics (cohort) by taking into account the specific requirements defined in their genome [12]. In this review, the use of nanomedicines as therapeutic agents will be discussed, specifically for the development of personalized therapies, and their application as novel treatments by the pharmaceutical companies. Although many types of nanomedicines exist, i.e., polymeric nanosystems, nanoparticles, magnetic nanoparticles, and liposomes, for example (Figure 2), all of them present common advantages among conventional therapies; which in general modify the pharmacokinetics and pharmacodynamics (pK/pD) of the active principles [23]. Specifically, these advantages can be grouped as follows (summarized in Table 1): (1) the size of the nanomaterials is very small, thus resulting in a large surface-to-volume ratio that is advantageous for the fine tuning of the nanomaterial’s surface; (2) activities of a different nature (lipophilic or hydrophilic) can be encapsulated in any type of nanosystem, thus enabling higher doses not possible in traditional therapies due to solubility problems; (3) they protect the encapsulated activities from the environment (e.g., light, nucleases); (4) they modify the pharmacokinetics of the active principles, allowing a controlled active release, which is advantageous to reduce the frequency of dosage and prolong the therapeutic activity; (5) they can be actively directed to the target organ, thus making possible a local therapeutic effect, increasing the therapeutic activity and reducing side effects; (6) the possibility of choosing among different nanosystem types gives nanomedicine the appropriate versatility to design the appropriate specific treatment to achieve a personalized therapy [4,12,15,17,18,19,20,26,27,30,31,32,33].

The pharmaceutical industry has perceived the numerous advantages of nanomedicines, and many studies are being carried out with the objective of reaching the market in the near future. However, although the basic research studies on nanomedicine are numerous, that does not correlate with the limited number of novel nanosystems that have been transformed into clinically commercialized nanomedicines nowadays [4,21,28,32]. In this context, this article attempts to analyze the current impact that nanomedicine research has on the pharmaceutical market and the added value that nanomedicines could give to the current therapies. The wide spectrum of diseases treated using nanomedicine will be analyzed, giving special attention to the field of personalized medicine. Although current nanotherapies are practically nonexistent in this field, it is expected that there will be an exponential increase in the number of studies and licensed products.

An optimistic point of view believes nanosystems are part of the 21st century industrial revolution, an enabling technology for the nations’ health and wealth enhancement. In this context, we are expecting nanomedicine for the personalization of treatments to offer appropriate therapies to a wider number of patients. On the other hand, a pessimistic point of view encourages a “go-slow” approach, taking a cautious position from which it is mandatory to first collect all information about nanomedicine’s risks (currently lacking), passing through regulatory agencies and finally approving only those fully characterized as safe therapies. As in many cases, the real situation is somewhere between those two points of view [34].

### 1.1. Nanomedicines in Pre-Clinical and Clinical Development: An Overview

**Figure 3:**
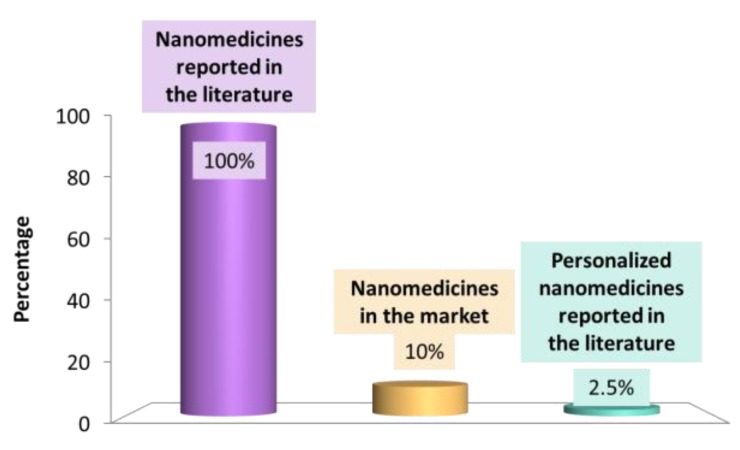
Plot representing the current nanomedicine world, where percentages correspond to literature available in 2017.

The number of scientific articles describing nanotherapeutics is huge: a search for the terms “medicine” and “nano” in Pubmed returns more than 15,000 articles. Nevertheless, the number of marketed nanomedicine products only represents one-tenth of them (Figure 3). In 2015 in the USA, only a few nanomedicines had been approved by the Food and Drug Administration (FDA), including: MyocetTM, Abraxane, Doxil, Eligard, Caelyx, DaunoXome, Genexol-PM, and Oncaspar (Table 2) [5,6,13,32,33]. The number of approved nanomedicines in the USA is similar to that corresponding to approved nanomedicines in Europe by the EMA. We will analyze the reason for this gap between the basic research and commercial use of nanotherapeutics and try to give some keys to enhancing the licensing of novel nanomedicines in the following sections.

The first FDA-licensed and marketed nanomedicine was the widely known Doxil in 1995, a <100 nm PEGylated liposomal nanosystem encapsulating doxorubicin, for the treatment of various types of cancers (e.g., metastatic breast cancer and ovarian cancer) [9,11,12,13,19,20,21,34,35], which is considered the dawn of nanotechnology being applied to medicine. In Europe, the first nanomedicine product approved by the EMA was AmBisome, a liposomal nanoformulation encapsulating amphotericin B for the treatment of fungal infections [4,7,34,36,37]. Since then, the number of nanomedicine studies has increased exponentially and, currently, papers and patents are numerous. The licensing of the first generic nanomedicine product by the FDA was for Lipidox, a generic version of Doxil, in 2013; some controversy is still present as to the safety and efficacy of both [38,39,40]. Among the type of nanomedicines under clinical trial, the vast majority are liposomes, although emulsions or hydrogels are also used [6,8]. Concerning the type of material composing the nanosystems—lipids, proteins, or polymer—drug conjugates are most often used to design nanomedicines intended to mainly treat cancer, since it is the one of the main causes of human disease and mortality in the 21st century in the developed countries [4,6,21,33,34], especially lung cancer, which has a high prevalence but a poor prognosis [41]. It is expected that by 2030, approximately 13 million people will die annually of cancer [9]; therefore, there is no doubt about the need to invest resources in finding novel therapies to fight it. Currently, there are more than 300 nanomedicines (mostly nanoparticles) under development to serve as anticancer agents and more than 20 nanomedicines approved by the FDA [4]. However, only a few of them will successfully become cancer treatments [13,20,28,31]. Among the advantages that nanomedicine can give to current therapies for cancer, the reduction of side effects due to the encapsulation of the active ingredients and their action in the target organs have to be emphasized. In addition, the use of nanocarriers can also help to circumvent the multidrug resistance effect, a drawback of current therapies [9].

Nanomedicines for cancer therapy are mostly designed by taking advantage of the enhanced retention and permeation effect (EPR effect), which enables carrier accumulation in the region of the tumor and permeation by passive diffusion, due to the high concentration gradient from the blood vessels [6,11,19,29,31,38,42]. Doxil is still one of the most used nowadays, but many other anticancer nanoformulations have been approved, such as Oncoprex, a lipid nanoparticle containing plasmid DNA that encodes for *TUSC2*, a tumor suppressor gene, in Phase II for the treatment of lung cancer in combination with Erlotinib; and Abraxane, albumin nanoparticles encapsulating paclitaxel for the treatment of various types of tumors (see Table 2) [7,12,32,33,34,43,44,45].

However, cancer treatment is not the only objective of personalized nanomedicine. Scientists and research organizations are aware of the requirements of novel, more efficacious treatments for a wide variety of diseases that affect many people. In the following, some examples of nanomedicines designed for specific diseases will be given.

The investigation of nanomedicines is also relevant to the field of neurodegenerative diseases, since current treatments lack efficacy, mainly due to a lack of knowledge of the pathogenic cascades involved in this group of diseases. In addition, neurodegenerative diseases occur in the central nervous system, which is protected by the blood–brain barrier; therefore, it is crucial that nanomedicines are designed in order to cross it and access the target organs [26,27].

Metabolic diseases also constitute an important group. Among them, diabetes is one of the most prevalent, for which efforts in the nanomedicine field have also been developed. Currently, diabetes therapy is invasive, which decreases patients’ compliance and leads to complications in their healthcare. For this reason, nanomedicine has been used to develop novel systems to deliver insulin in a less invasive way. This system, composed of polymeric nanoparticles such as the Smart Insulin L-490, from Merck, can be defined as a kind of personalized nanotheranostics, since it detects the level of patient glucose and responds to this stimulus by releasing more or less insulin [46].

Nanomedicines can also be applied to the treatment of rare diseases. A rare disease is defined in the United States as a condition that affects fewer than 200,000 people and, in the European Union, when it affects fewer than 1 in 2000 people [47]. A rare disease is also known as orphan disease because drug companies are not interested in adopting them to develop treatments. The number of studies devoted to treat rare diseases using nanotherapeutics is limited, since pharmaceutical industries assume that the costs required to develop them will not produce enough economic benefit. For this reason, efforts to improve and bring to market treatments for rare diseases are coordinated by the Medicine Agencies (FDA, EMA), which provide incentives for drug companies to develop treatments for rare diseases; currently, there are some nanodrugs under development [19,48,49,50,51]. An example of a nanomedicine for rare disease treatment could be the drug Lysodase, a poly-(ethylene glycol) (PEG)–glucocerebrosidase nanoconjugate that can be used for chronic enzyme replacement in patients with Gaucher’s disease [48].

Finally, there is a group of diseases that has sometimes been defined as “neglected diseases” since it comprises diseases with poor visibility and low political impact, such as tuberculosis, malaria, leprosies and dengue virus infections, diseases that usually affect developing countries and are also known as “diseases of poverty” [52,53]. For this reason, public and private organizations from the developed countries have not traditionally invested in research into new treatments. Instead, efforts have been devoted to the study of nanomedicines for those diseases affecting wide groups of people in developed countries [52,54]. Nevertheless, researchers studying these diseases have seen nanomedicine as a promising alternative to develop efficient treatments. Although the number of studies devoted to these diseases is limited, some advancement in the nanotherapeutics for neglected diseases already exists [53]. Malaria is one of the most widespread diseases in Africa, due to the rapid transmission by mosquitoes, the resistance of most *Plasmodium* strains to available drugs and poor drug solubility and bioavailability, which leads to the administration of huge drug doses that usually cause toxicity. The encapsulation of the actives in nanocarriers has proven advantageous, such as the encapsulation of primaquine in liposomes and solid–lipid nanoparticles [53]. Much more effort is required before these products reach the market.

Tuberculosis is a disease affecting more than eight million people each year, mainly due to the multidrug resistance effect of *Mycobacterium tuberculosis*. Current treatments require repeated administration over long periods of time, which usually produces severe side effects. The advantages that nanomedicine could give are: reduction of drug dosage, decreased frequency of administration, and amelioration of side effects. However, these treatments are not already in clinic due to issues in selecting the administration route (oral vs. pulmonary) to enhance drug bioavailability [25,53].

Although there are numerous references in the literature to nanotherapeutics and current existing nanomedicines, it is fair to admit that we have not yet succeeded in developing efficient and curative therapies for many diseases. The main drivers of failure could be our misunderstanding or forgetting of the existent heterogeneity in diseased individuals. In addition, current nanotherapeutics can be defined as first-generation therapies, without specific targeting to the desired site of action. Therefore, we have not been able to fine-tune nanomedicines in accordance with the specific requirements of each patient and much effort is required to develop scientific platforms of knowledge of patients’ variability to enable the development of personalized nanomedicines.

### 1.2. Challenges in Personalized Medicine

Personalized nanomedicine involves the prediction, treatment, and prevention of diseases on an individual basis. It consists on the design of therapies matching a specific treatment to a specific patient or group of patients to achieve a positive outcome [4]. The origin of personalized medicine was the birth of “omics” (i.e., pharmacogenomics, pharmacoproteomics and pharmacometabolomics) in the early 2000s, which enabled the study of the specific genetics of each individual [12]. As described in the last section, the number of nanomedicine products is huge, but not a single one is designed as a personalized medicine (Table 1) due to the reduced number of publications concerning personalized nanomedicine (Figure 3). A theoretical approach ensures that personalized therapies may be designed by taking advantage of nanomedicines, as described in Table 1 [38].

This lack of current nanomedicine products for personalized medicine can be attributed to many factors. On the one hand, there are nanomedicine **formulation issues**. As stated above, current nanotherapeutics are first-generation therapies without active targeting to the desired organ, such as Doxil, which is expected to reach tumors by the EPR effect [38]. This formulation does not include an active targeting moiety; therefore, it is not directed to a specific cell type or organ. This lack of active targeting could be considered a formulation issue that needs to be circumvented. Nevertheless, Doxil has been used for the treatment of many tumors along the years, but it must be taken into account that each tumor and even each region inside a sole tumor has different environment conditions (e.g., stromal cells, hypoxic gradient, fenestration, and extracellular matrix). Therefore, novel second-generation nanotherapies should include a targeting moiety in their formulation. The most used targeting moieties have been monoclonal antibodies (e.g., OX26 to target the transferrin receptor, overexpressed in blood–brain barrier cells [55]), since they target specific molecules expressed by a single type of cells. Other type of tunable nanosystems that could be useful for personalized therapies are viruses and bacteriophages [3], such as the currently extended oncolytic virotherapy (e.g., the design of novel treatments to fight against pancreatic adenocarcinoma by taking advantage of adenoviral vectors [56]), since they can be genetically modified to express or suppress a gene of interest [38]. It is also important to keep in mind that to achieve the personalization of a therapy, the use of gene material as the active principle is recommended as compared with pharmacological therapies with drugs. Therefore, researchers should direct their investigations towards gene therapies instead of drug therapies, since the latter are most often used in current nanomedicines.

**Economic issues** are another challenge to circumvent before the success of personalized nanomedicine can be guaranteed. The entire process of commercializing a novel nanodrug is estimated to last for more than 10–15 years and cost around $1 billion [6]; the cost is higher in the case of personalized medicine. It is reasonable to assume that it is neither realistic nor efficient to design personalized treatments for a single patient with current technologies; it must be a key research objective to find groups of patients with common characteristics so as to create personalized therapies for more than a single patient [29].

As a consequence, novel research on personalized nanomedicine should start by defining molecular profiles, showing the gene expression characteristics of each patient and, even more importantly, the patterns that are expressed over time and as a function of the progression of a disease. From this basic knowledge, clinicians could define the stage and progression of the disease and develop nanotherapies specific for each gene pattern [4]. This rationale has been demonstrated to be useful in the case of metastasis from a local tumor. As an example of this approach, the group of Golub demonstrated that the tumor response to a treatment was dependent on tumor stroma [57,58]. From these results, it was concluded that metastasis was dependent not only on tumoral factors, but also on stromal and inflammatory factors [4]. Therefore, before using and even designing nanotherapy for a cancer patient, his/her genetic and biological characteristics must be carefully studied to achieve efficacy [31].

## 2. Development of Novel Nanomedicines

From the experience on already designed and marketed nanotherapeutics, it is expected that nanomedicine will revolutionize current therapies, but to achieve it pharmaceutical industries must believe in the benefits of nanomedicines. Currently, there is a huge gap between research laboratories and clinical use of nanomedicines, even although there are an enormous number of publications regarding nanotherapeutics. To encourage societal acceptance of nanomedicine as a therapy of the future, experiments must be carefully designed to facilitate its translation to the market and its use in personalized therapies.

### 2.1. Design of Nanomedicines in Research Labs

It is recommended to design nanosystems using the simplest procedure/platform possible, and taking into account the ADME parameters, since this is fundamental to ensuring the safety and efficacy of the nanomaterial (absorption, distribution, metabolism, and elimination) [7,33]. An efficient methodology to perform the physicochemical characterization of nanomaterials is applying chemical manufacturing and control (CMC) practices to ensure the quality of the nanoproduct, although CMC practices applied to nanomedicine products do not already exist [33].

To efficiently design novel nanoformulations, there are many aspects that must be taken into account, since it is a multidisciplinary discipline, including professionals of diverse areas, such as physicists, chemists, biologists, and clinicians [11]. These aspects can be grouped as follows: (1) issues concerning nanosystems’ properties; (2) issues concerning nanotoxicity; and (3) issues concerning heterogeneity between patients.

First of all, the properties of the nanosystems themselves must be well defined: the material type and molecular weight, the hydrophobicity, shape, surface charge, and nanomaterial size; active molecules and the presence of functionalizing molecules [4,12,20]. These properties have been highlighted in many studies, although only a few have characterized what is most important before preclinical and clinical studies [14,38]. In addition, they have been extrapolated from the properties of the bulk materials, although they usually differ [4,5,7]. To achieve specific targeting of the nanosystems to the desired target organs, functionalizing molecules are used. Currently marketed nanoproducts are defined as first-generation nanosystems since they lack these functionalizing molecules and expect nanocarriers to passively reach the tumors by taking advantage of the above described EPR effect (e.g., Doxil, as discussed above) [11,19,29,38,42]. In next-generation nanomedicines, it is strongly recommended to add targeting when designing the nanomedicine [4]. Although the most widely known is the active targeting using a concrete molecule that binds to a specific receptor of a single type of cell (e.g., monoclonal antibodies), the targeting can also be physical, using targeting molecules that respond to stimulus, such as the use of magnetic nanoparticles, which can be useful for thermal therapies in the case of tumors: the microenvironment of the tumor has a higher temperature than the body, thus heating magnetic nanoparticles and producing toxicity in the tumor [10,23]. Nevertheless, for personalized therapies, the use of active targeting moieties is strongly encouraged, since the targeting can be much more specific than using the physical targeting, although the combination of both could be also useful [6]. BIND-014, or docetaxel-loaded polymeric nanoparticles, is an example of an nanodrug actively targeted to prostate tumor cells; in Phase II clinical trials, it specifically targets the prostate-specific membrane antigen (PSMA) [11,33,59].

Another property of nanosystems that we would like to remark on is their interaction with biological components. The addition of a poly(ethylene glycol) (PEG) layer, after nanoparticle formation or as an excipient to form the nanoparticles, has been widely reported in the literature to decrease non-desired protein interactions, since it enhances the “stealth effect” of nanomedicines: the increase of their blood half-time, thus favoring the EPR effect [12,18,31,35]. Although they seem to be useful, usually nanomedicines are characterized in an artificial environment, namely in PBS media, without taking into account what they will be surrounded by. Most of the existent nanomedicines are designed for intravenous administration (Table 1); therefore, they will be suspended in the blood, a solution full of cells and proteins. Nanosystems will interact with biological components, and their fate and final therapeutic action will depend on these interactions [12]. There are many reviews describing these interactions [15,60,61,62]; although a full discussion is outside of the scope of the present review, being careful about this aspect is recommended.

When nanosystems come into contact with biological fluids (e.g., blood, interstitial fluid, or mucus), they are coated with proteins that usually vary in terms of their surface properties (e.g., charge, hydrodynamic size). The protein that usually binds most strongly is albumin, since it is the majority protein of the blood, but immunoglobulins, fibrinogen, apolipoproteins, and proteins from the complement cascade are also present [12,15,63]. A fundamental comprehension of the interactions between nanomaterials and body proteins and cells is necessary. However, the nanomaterial–protein interactions can be exploited to study biodistribution and guide the nanoparticles to specific tissues. This is the case with albumin-coated paclitaxel (nab-PTX) and the interaction with SPARC (secreted protein acidic and rich in cysteine), overexpressed in increased tumor invasion and metastasis: SPARC-positive patients responded better (83%) to nab-PTX than SPARC-negative patients [12,64]. Decreasing the immunogenicity of a nanomaterial is also of critical importance: an understanding of the immune reactions to therapeutic and diagnostic nanomaterials is required to determine which characteristics warrant repeated parenteral administration without adverse reactions. Further understanding of the interactions between proteins and nanomaterials is required to establish their potential for personalized medicine. People display individual differences in protein circulating levels. This variability can explain the differences in a patient’s response to nanomedicines or their higher susceptibility to side effects [12].

Understanding nanomedicine–biological component interactions could allow the design of personalized nanocarriers. The utilization of intracellular enzymes to trigger nanomedicine’s therapeutic activity has implications for personalized medicine. The designed nanomedicines could be more beneficial to a certain cohort of patients if they are designed taking into account these differences [12].

It is also remarkable that a single nanomedicine type can be designed as a multifunctional system, to act as a nanotheranostic nanocarrier. This is the case with magnetic nanoparticles encapsulated in polymeric nanoparticles also loading a chemotherapeutic drug; they are used as diagnostic agents to visualize tumors through magnetic nanoparticles, but also as therapeutic agents to attack tumoral cells [4,65,66,67,68].

The second issue to take into account is the nanotoxicity [4,12]. Currently, toxicity type studies have been adapted from those traditional toxicity studies in macromolecules, but they should be reformulated to consider the particularities at the nanoscale [38]. Although many research works exist concerning toxicity issues at the nanoscale, currently toxicological studies at clinical stages and regulations on how to test the toxicity of nanomaterials, their degradation, and their accumulation in the body when designing them do not exist [12]. However, there are some organizations that give recommendations for the design of safe therapies. In Europe, the ETPN remarks on the importance of toxicity reduction in order to convince investors and promote the business of nanomedicine.

A third issue to take into account is the heterogeneity between biological characteristics of individuals; even inside a tumor, the vasculature is heterogenous. Therefore, when administering nanomedicines, absorption and distribution can present a lack of uniformity [31]. Only as an example, concerning the immunology system, many efforts have to be developed for the personalization of therapies. It is known that different patients respond differently to a common antigen. For example, the activation of the complement leading to the C activation-related pseudo-allergy (CARPA) effect has been demonstrated to be different from patient to patient [7,40]. Therefore, during the design of nanomaterials’ surfaces, these individual differences must be taken into account to explain the slight variations between the specific requirements of each group of individuals.

Added to this heterogeneity, and sometimes underestimated, is the fact that most basic research nanomedicine studies are not designed to treat a specific disease affecting a concrete group of patients. However, to design a success therapy, it is key issue to keep in mind the target disease and patients [7,20]. For example, in a tumoral microenvironment (as well as in ischemia, inflammation, arthritis, and atherosclerosis), the pH is acidified as compared to the rest of the body due to the production of glycolic, carbonic, and lactic acid by cancer cells, which leads to cancer invasiveness and aggressiveness [69,70,71,72]. Therefore, specific physical targeting of a tumor could be performed by a pH-sensitive polymer, protein, surfactant, or lipid that is stable at a physiological pH (in most of the body) but is destabilized upon pH decrease, thus promoting the release and cellular internalization of the encapsulated compounds [23,73].

Last but not least is the patient’s acceptance of the therapy. More than 70% of currently available nanoproducts consist of intravenously administered products, which are not patient-friendly, thus reducing patients’ adherence to treatment and negatively affecting the efficacy [8]. This aspect should be taken into account from the very beginning of the design.

### 2.2. From Lab to Market

Once a novel nanomedicine has been designed and studied at a research lab, it is time to translate that knowledge to the pharmaceutical industry to achieve final commercialization after the clinical study. While nanomedicine holds promise, many challenges need to be overcome to achieve its full potential. The translation to the market has traditionally been a hard step for any novel medicine; it is especially complicated and expensive for nanomedicines, as compared to small molecules [10,34,54]. It is worth noting the example of BioNTech company products. Their pipeline includes many nanovectors designed for the personalization of therapies, specifically focused on the use of codifying mRNAs as cancer vaccines (immune-oncotherapy), typically to fight melanoma and breast cancer. IVAC mutanome vaccine is the most representative nanoproduct that is already undergoing Phase I trials. It can be defined as an individualized medicine since the codifying mRNA it contains is designed individually regarding the specific expression of antigens of a single patient or a group of patients [74,75,76]. However, examples like BioNTech are difficult to find, since, from a commercial perspective, personalized nanomedicine only plays a marginal role as compared to traditional therapies, and the number of patented products is still low (less than 1% of total drug patents per year) [77]. In addition to the points indicated in Section 1, in this section, a deeper analysis of the factors that must be overcome to reach the market will be presented. These factors can be grouped as follows: (1) technical issues; (2) lack of regulations; and (3) economic risks.

Concerning technical issues, first of all, it must be taken into account that the nanoworld is difficult to understand, since it is influenced by interactions with the environment; and it is common to obtain discordant results between in vitro and in vivo models. For this reason, both cellular and animal models must be carefully selected to represent the expected behavior in humans [12,20].

Bottom-up synthetic strategies that consist on building up the nanomaterial from molecules are strongly recommended for the development of pharmaceuticals instead of top-down approaches, since they are more cost-effective and enable a higher degree of precision [4].

One of the major problems in reaching the market is the scaling up of the synthesis process [7,12,54,78]. Although controlled at the laboratory scale, in medium- and large-scale production, other factors affect the process, and batch-to-batch variations appear in nanomedicines’ characteristics that prevent clinical testing [10,38]. In recent years, microfluidic systems have been gaining importance since they enable the large-scale production of nanoformulations under a controlled process [18]. In addition, nanomedicines’ scale-up usually entails a high cost that can hamper the success of the novel therapy; for this reason, the cost-effectiveness of nanomedicines’ scale-up must be taken into account from the very beginning of the design process [54].

Other technical issues are mainly related with freeze-drying, synthesis with GMPs, sterilization, and storing processes. Concerning **freeze-drying**; it has traditionally been a limiting step for the synthesis of any kind of drug, and this problem is more remarkable at the nanoscale. Freeze-drying must be carefully set up to ensure the physicochemical properties of nanosystems are maintained after the freeze-drying and avoid possible degradation/aggregation of nanomaterials [7].

Concerning **sterilization**, it is a must that all products administered to humans are synthesized under good manufacturing practices (**GMPs**) to ensure their sterility and the absence of pathogens [7,38]. The sterilization process can also be a difficult step to set up. Both sterilization and synthesis, specifically under GMPs, produce a notable increase in process costs that pharmaceutical industries are not always willing to take on if they do not envisage a real benefit [12].

The **storage** of nanomedicines could also represent a bottleneck. Usually, in research labs, nanomedicines are used just after they are synthesized, since their stability could be reduced over time. To prolong the storage time, the most common mechanism is the freeze-drying of nanomaterials, which, as stated above, could be difficult. Therefore, storage represents a step forward [12].

Second, we have to take into account that current specific legislation or guidelines regarding nanomedicines is missing [10,34], which produces an uncomfortable uncertainty that discourages pharmaceutical industries from enrolling in nanomedicine. Regulation issues will be further described in the next section.

**Figure 4:**
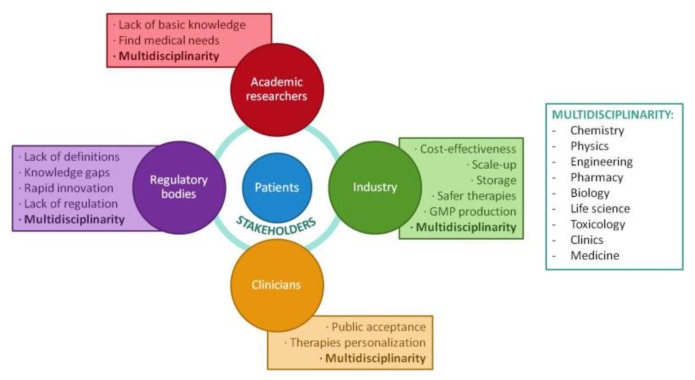
Schematic representation of the stakeholders involved in the translation of nanomedicines from the lab to the market and the most common challenges involved for each of them.

Related to regulation, there exist economic risks. Although there are initiatives for public–private partnership in the development of novel nanomedicines, private industry does not see an appropriate opportunity for their implantation, so they have not already succeeded [52]. There is an urgent need for effective communication between research labs, public initiatives, and all internal and external stakeholders (e.g., clinicians, investors, etc.) involved in each project, applying risk-mitigation strategies to demonstrate the cost-effectiveness (Figure 4) that makes nanomedicine different or relevant compared to current therapies, therefore seeing it as a cutting-edge technology for the treatment of human healthcare and the improvement of a country’s economy [7,19,54,78,79]. At this point, it is important to highlight the problem of risk aversion from industries and economic stakeholders, which prevents nanomedicines from even starting to be developed [34]. Since financial stakeholders (investors) are the key to reaching the market, we should change their perspective so they feel sure about investing in nanomedicine research. To achieve this milestone, they should be provided with a wide knowledge from the point of view of the many disciplines involved in a nanomedical study (Figure 4). In parallel, nanomedicine knowledge must also be expanded in the non-scientific community. Only by divulging scientific knowledge to all involved stakeholders to make them understand the importance of the nanoworld in future therapies will the “nanorevolution” be possible [34,54].

**Figure 5:**
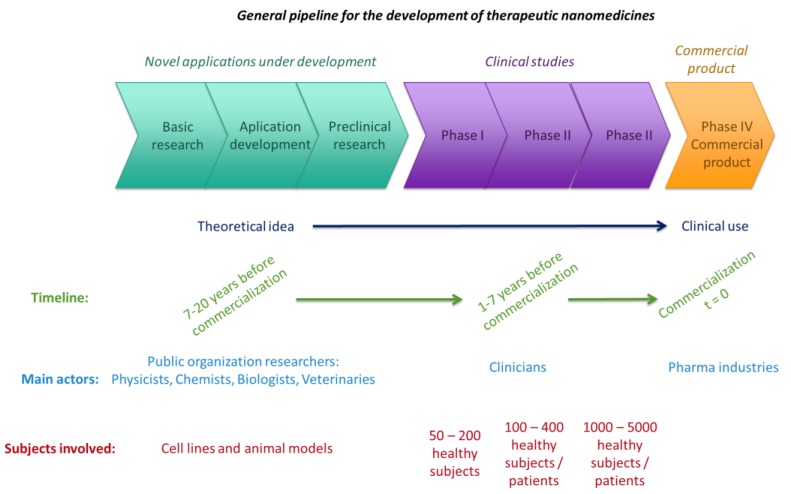
Schematic representation of the process of nanomedicine development over time. Modified from [7,8].

Therefore, some hurdles to be circumvented by the pharmaceutical industries relate to demographic trends and changes in healthcare priorities, the minimization of the economic and health risks related with nanotherapies, the impact of novel therapies on society, and conveying scientific knowledge to patients [4]. In this context, the concept of risk sharing schemes appeared [80]. It refers to the novel agreements between consumers and the pharmaceutical industry, in which the uncertainty over new treatments is regarded. In these schemes, the relationship between costs and benefits for both players is balanced to avoid the release of new drugs that are not well-characterized or are even cost-ineffective. In the near future, therapies must progress towards more personalized treatments, which will force pharmaceutical business models to adapt. In addition, the manufacture of nanomedicines is substantially more expensive and subjected to more intricate quality control than small molecule synthesis, a factor that contributes to the low investment of large pharmaceutical companies and to the elongation of each phase of the development process (Figure 5) [11].

## 3. Pharmaceutical Industry Opportunities

A few years ago, nanomedicine arose as a novel therapeutic technique with expected social benefits; it promises a cure for many diseases, especially cancer [4,41]. However, this has not happened; although substantial efforts have been made to enhance the efficacy of nanotherapeutics to fight cancer, meaningful clinical benefits have not already been achieved, and many more studies are required to reach that point [31]. First of all, due to the risks to human health and the uncertainties associated with these risks, it is difficult to earn consent for clinical trials [12]. Another pitfall is the lack of knowledge of how molecular mechanisms govern some diseases. Both factors, the risk and the lack of knowledge, contribute to the public’s perception of doubts and controversies, which is not a favorable situation in which to get the pharmaceutical industry involved. It is important that clinicians believe in nanomedicines so that they can transmit their confidence about safety to patients, who trust them [4].

In order to reduce the uncertainty, Real World Data (RWD) studies appeared, which summarize the results from clinical trials with real patients, in which a previous statistical analysis was performed to discuss all existent clinical data, including characteristics of each patient cohort (e.g., comorbidities, ae, gender, etc.) [81]. Thus, RWD can be a useful tool for personalized nanomedicine, taking advantage of a great variety of clinical trials over the world. RWD can provide data from all stakeholders involved in new nanomedicine product development and commercialization processes, to effectively manage all the steps of the process, reducing healthcare delivery costs [81].

### 3.1. Regulations of Nanomedicines

Currently, the regulatory field is not yet mature: the development of nanomedicines has evolved faster than the regulations [34,78], and there is still no specific regulation regarding nanomedicine in the European Union [4,7,10,78]. Regulatory reform is required for nanomedicine to establish “nanoguidelines” in order to facilitate their translation into commercial products, since traditional regulations are not appropriate for nanoproducts [7,12]. For this reason, the medical use of nanosystems is currently at a very early stage, since most nanodrugs are in different stages of pharmaceutical development, without having arrived on the market yet. Accordingly, the development of techniques to confirm the safety of these novel nanomedicines has not been achieved either [10].

However, the vision of nanomedicine development must be optimistic, since huge efforts are prone to be successful in the near future. Based on existing nanomedicine, the trajectory leads towards success. Only Doxil has a market value of more than US$600 million in annual sales [35]. The United States was the first country to start an official government program to fund nanotechnology research in 2000, reaching a budget of around USD$1.5 billion last year [19]. It is also worth noting the work of organizations, mainly those devoted to research into cancer treatments, such as the National Cancer Institute (NCI), which, with the help of the FDA, is standardizing a protocol assay cascade for total preclinical toxicology, pharmacology, and efficacy characterization of nanomedicines. This protocol is available on their website [6,82], to be followed by nanomedicine researchers. In addition, NCI has established the Nanotechnology Characterization Laboratory (NCL), which offers assay cascade characterization for the nanosystems they consider real candidates to reach the market [6,7,8,35].

In parallel, Europe has created the ETPN as a part of the Horizon 2020 program, which intends the creation of a European NCL-like laboratory, although Europe prioritizes translational medicine [19]. Although the approved number of nanomedicines in Europe is slightly lower than in the USA, this initiative is very important to arrive at higher commercialization levels due to common guidelines and quality controls of novel nanomedicines [38]. In both regions, Europe and the United States, many consortiums have been created following the example of NCL and ETPN and under their advice and guidance. They serve to control the quality of nanomedical research, helping companies and researchers to reach commercialization [7,19]. This is an encouraging development, since consortiums represent places where different types of specialists join together to discuss the requirements that nanomedicines must fulfill to reach the market, from early study of disease biology to the nanomedicine approach to treat it and the translatability of the production process [7,20,27].

At this point, certain controversies exist in the scientific community among those who think that the translation from labs to the market is too cautious and those who think that we must be more cautious [4,35].

Although they are not regulated by any legislation, ethical aspects must also be taken into account when designing novel formulations [4]. Specifically, in the case of personalized medicine, patients’ genetic information is required to design personalized therapies. Healthcare professionals must manage the data to enable the design of personalized therapies, but with the confidentiality and privacy that patients deserve [12].

### 3.2. The Specific Case of Personalized Nanomedicine

**Figure 6:**
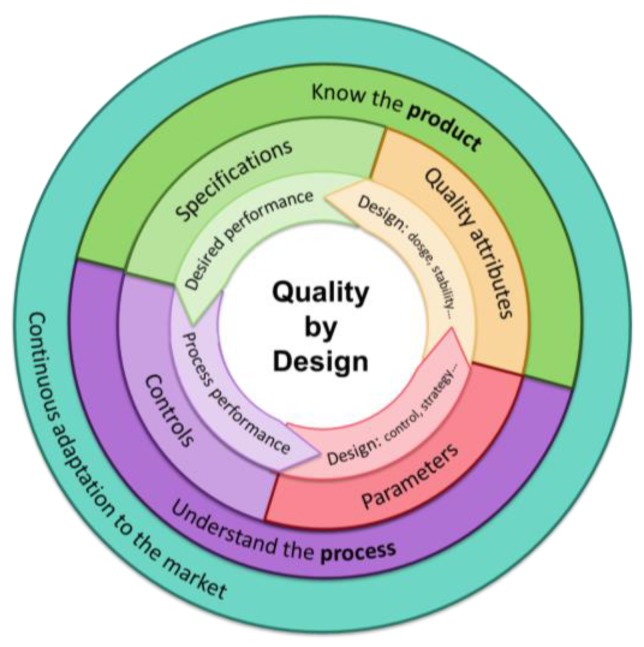
Schematic representation of the Quality by Design approach, recommended by the FDA for the development of novel nanomedicines. Adapted from [83].

In parallel to the requirements for all nanomedicines in general, personalized nanomedicine requires other specific criteria to have an impact on clinical therapies [12,35,54]. Nanomedicine is a cutting-edge research field that, in a near future, will represent significant and technologically promising advancements, with a high potential to lead to a transformation of the available therapies [8]. However, currently there exist many challenges that must be addressed before it can reach its full potential [12]. Nanomedicines have different properties than the bulk materials they come from; therefore, these properties must be studied when nanomaterials are formed, using appropriate characterization methods that usually differ from the methods used at a larger scale [35]. When specifically designed as a personalized therapy, the associated risks due to the uncertainty caused by the lack of experience in the field are more noticeable than in other diseases [12]. In the case of cancer, for example, image-guided personalized therapy is strongly recommended since it enables the detection of specific biomarkers to design a nanosystem specifically targeted to the ligands found for each patient [4]. However, personalized therapy is designed for the specific requirements of an individual; thus it is not safe for all patients, which produces uncertainty in investors and difficulties in receiving approval from regulatory agencies, since specific regulations for each personalized nanotherapy do not already exist [7,12]. Quality by design strategies (Figure 6), which consist of a continuous improvement of the product taking into account the requirements of the market and the parameters of safety, quality, and efficacy [4], are encouraged by the FDA and notably appropriated for personalized nanomedicines, since they enable the design of a nanomedicine with properties that will evolve during the development process, taking into account the necessities of each cohort of patients. These strategies enable a reduction of production costs, since they start from the design of a single nanomedicine that can be adapted to a group of patients (personalized) during the development process [4].

From the point of view of the personal biology of each individual, many differences exist due to the complexity of biological processes [12]. Although patients’ variability and heterogeneity is a huge hurdle for personalized therapies, alternatives exist for the development of personalized nanotreatments for groups of patients with similar characteristics. The versatility of the nanoscale treatments (e.g., various targeting at the surface of a common nanoparticle) enables the design of libraries of a single nanoproduct with many slight variations, with each variation appropriate for a group of patients.

Personalized therapies, therefore, imply an increase in production costs, which may not be acceptable for the pharmaceutical market. The pharmaceutical industry needs to refocus their strategic lines to be adapted to personalized medicine [35,54], which is usually designed to treat a specific cohort of patients, thus increasing the associated risks and challenges. However, it is important to emphasize the positive gain-to-risk (cost-effective) relationship to demonstrate that personalized therapies can be really useful and necessary. As stated above, nanomedicine is specifically appropriate as a personalized therapy due to its versatility and ability to transfer knowledge (e.g., stability, toxicity, interaction with blood proteins) from one nanosystem to another easily. These treatments are expected to deeply impact on treatments for orphan diseases, since no current therapies exist [12].

## 4. Conclusions

Nanomedicine has emerged as a promising treatment for many diseases, as numerous examples of current nanomedicine treatments support. However, the number of licensed products is still limited as compared to research studies. This is specifically notable in the field of personalized medicine, where no licensed nanoproduct exists. However, the scientific community is optimistic. Many consortiums have been created and are working in close relationship with regulatory agencies to support the translation of nanomedicine research lab results to the pharmaceutical industry with the goal of the commercialization of novel nanomedicines. They are trying to establish regulations and guidelines to circumvent the current challenges of personalized nanomedicine so that it can become the medicine of the future.

## Figures and Tables

**Table 1:** Summary of the main advantages of personalized nanomedicines to become the medicine of the future and the specific sub-properties for its application to personalized nanomedicine.

Personalized Nanomedicines
Nanometric scale dimensions
Tunability/versatility
Use of labile compounds (e.g., siRNA)
Active principles: encapsulation and protection
Modification of actives pharmacokinetics
Specific organ targeting moieties
Arrival to specific cellular compartments
Adaptation to the requirements of a cohort of patients
Adaptation of therapy patterns to each patient (e.g., dosage, frequency, etc.)

**Table 2:** Examples of nanomedicines that can be currently found in the industrial pharma development/sales.

Drug Name (Tradename/Active Principle)	Company	Type of Nanoformulation	Indication/Route of Administration	Status
Abraxane/Paclitaxel	Abraxis (Warminster, Penn State, USA) and Celgene (Summit, New Jersey, USA)	Albumin nanoparticles	Various cancers/IV	Marketed
Adcetris/Brentuximab	Seattle Genetics (Bothell, Washington, USA)	Antibody-drug conjugate	Non-Hodgkin lymphoma/IV	Marketed
ALN-TTR02 (Patisiran)/siRNA	Alnylam Pharmaceuticals (Cambridge, Massachussets, USA)	Liposome	Transthyretin amyloidosis/IV	Phase II
AmBisome/Amphotericine B	Astellas Pharma (Chuo, Tokio, Japan)	Liposomes	Fungal infections/IV	Marketed
Aurimune/---	Cytimmune sciences (Rockville, MD, USA)	Colloidal gold	Solid tumors/IV	Phase I/II
Auroshell/---	Nanospectra Biosciences (Houston, Texas, USA)	Gold-silica nanoshells	Lung cancer/IV	Phase I
BIND-014/Docetaxel	Bind Therapeutics (Cambridge, Massachussets, USA)	Polymeric NPs	Solid tumors/IV	Phase II
Caelyx/Doxorubicin	Janssen (Beerse, Belgium)	PEGylated liposome	Solid tumors/IV	Marketed
DaunoXome/Daunorubicin	Galen Limited (Portadown, United Kingdom)	Liposome	Solid tumors/IV	Marketed
Diprivan/Propofol	AstraZeneca (London, United Kingdom)	Nano-emulsion	Anesthetic/IV	Marketed
Doxil/Doxorubicin	Janssen (Beerse, Belgium)	PEGylated liposomes	Various cancers/IV	Marketed
Eligard/Leuprorelina	Tolmar (Fort Collins, Colorado, USA)	PEGylated polymeric NPs	Prostate cancer/IV	Marketed
Emend/Aprepitant	Merck (Darmstadt, Germany)	Nanocrystal	Anti-emetic/Oral	Marketed
Genexol-PM/Paclitaxel	Samyang Biopharm (Seongnam, South Korea)	PEG-PLA polymeric micelles	Various cancers/IV	Marketed
Invega sustenna/Paliperidone palmitate	Janssen (Beerse, Belgium)	Nanocrystal	Schizophrenia/IM	Marketed
Ivac-MUTANOME/specific mRNAs	BioNTech (Mainz, Germany)	mRNA vaccine	Breast cancer and melanoma/Intranodal	Phase I
L-490/Insulin	Merck (Darmstadt, Germany)	Polymeric nanoparticles	Diabetes type I/SC	Phase I
Lipotecan/Camptotethin	Taiwan liposome (Taipei, Taiwan)	Polymeric micelles	Various cancers/IV	Phase I/II
Marqibo/Vincristine	Talon therapeuticals (Mississauga, Ontario, Canada)	Sphingomyelin -based liposomes	Leukemia and melanoma/IV	Marketed
Megace ES/Megestrol	Par Pharmaceuticals (Woodcliff Lake, New Jersey, USA)	Nanocrystal	Anti-anorexic/Oral	Marketed
Myocet/Doxorubicin	Teva Pharmaceuticals (Pétah Tiqvà, Israel)	Liposome	Various tumors/IV	Marketed
NC-6004/Cisplatine	NanoCarrier Co. (Kashiwa, Chilba, Japan)	Micelle	Lung cancer/IV	Phase I/II
Oncaspar/Pegaspargase	Enzon Pharmaceuticals (Farmingdale, New York, USA)	Polymer-protein conjugate	Acute Lymphoblastic leukemia (ALL)/IM or IV	Marketed
Oncoprex/pDNA TUSC2	GenPrex (Austin, Texas, USA)	Liposomes	Lung cancer/IV	Phase II
Onivyde/Irinotecan	Merrimack Pharmaceuticals (Cambridge, Massachussets, USA)	PEGylated liposomes	Pancreatic metastatic cancer/IV	Marketed
Ontak/Denileukin diftitox	Seragen (Madrid, Spain)	Protein NPs	Various tumors/IV	Marketed
Rapamune/Rapamycin	Wyeth/Pfizer (Philadelphia, Penn State, USA)	Nanocrystal	Immunosuppressive/Oral	Marketed
Tocosol/Paclitaxel	Oncogenex Technologies (Bothell, Washington, USA)	Nano-emulsion	Various tumors/IV	Marketed
Tricor/Fenofibrate	Abbott (Chicago, Illinois, USA)	Nanocrystal	Hypercholesterolemia/Oral	Marketed
---/siRNA PCSK9 synthesis inhibitor	Alnylam/Tekmira (Cambridge, Massachussets, USA)	Lipid NPs	Hypercholesterolemia/IV	Phase I
---/siRNA transthyretin inhibitor	Alnylam/Tekmira (Cambridge, Massachussets, USA)	Lipid NPs	Amyloidosis/IV	Phase II

IV: Intravenous; NPs: nanoparticles; PEG: poly(ethylene glycol); PLA: poly (lactic acid).

## References

[1] Duncan R., Gaspar R. (2011). Nanomedicine(s) under the microscope. Mol. Pharm..

[2] Gaudin A., Andrieux K., Couvreur P. (2015). Nanomedicines and stroke: Toward translational research. J. Drug Deliv. Sci. Technol..

[3] Sunderland K.S., Yang M., Mao C. (2017). Phage-Enabled Nanomedicine: From Probes to Therapeutics in Precision Medicine. Angew. Chem. Int. Ed..

[4] Ge Y., Li S., Wang S., Moore R. (2014). Nanomedicine.

[5] Weissig V., Guzman-Villanueva D. (2015). Nanopharmaceuticals (Part 2): Products in the pipeline. Int. J. Nanomed..

[6] Bobo D., Robinson K.J., Islam J., Thurecht K.J., Corrie S.R. (2016). Nanoparticle-Based Medicines: A Review of FDA-Approved Materials and Clinical Trials to Date. Pharm. Res..

[7] Bazile D.V. (2014). Nanotechnologies in drug delivery—An industrial perspective. J. Drug Deliv. Sci. Technol..

[8] Etheridge M.L., Campbell S.A., Erdman A.G., Haynes C.L., Wolf S.M., McCullough J. (2013). The big picture on nanomedicine: The state of investigational and approved nanomedicine products, Nanomedicine Nanotechnology. Biol. Med..

[9] Cerqueira B.B.S., Lasham A., Shelling A.N., Al-Kassas R. (2015). Nanoparticle therapeutics: Technologies and methods for overcoming cancer. Eur. J. Pharm. Biopharm..

[10] Bregoli L., Movia D., Gavigan-Imedio J.D., Lysaght J., Reynolds J., Prina-Mello A. (2016). Nanomedicine applied to translational oncology: A future perspective on cancer treatment, Nanomedicine Nanotechnology. Biol. Med..

[11] Brown P.D., Patel P.R. (2015). Nanomedicine: A pharma perspective, Wiley Interdiscip. Rev. Nanomed. Nanobiotechnol..

[12] Zhang X.Q., Xu X., Bertrand N., Pridgen E., Swami A., Farokhzad O.C. (2012). Interactions of nanomaterials and biological systems: Implications to personalized nanomedicine. Adv. Drug Deliv. Rev..

[13] Weissig V., Pettinger T.K., Murdock N. (2014). Nanopharmaceuticals (part 1): Products on the market. Int. J. Nanomed..

[14] Fornaguera C., Solans C. (2017). Methods for the In Vitro Characterization of Nanomedicines—Biological Component Interaction. J. Pers. Med..

[15] Hall J.B., Dobrovolskaia M.A., Patri A.K., McNeil S.E. (2007). Characterization of nanoparticles for therapeutics. Nanomedicine (Lond.).

[16] Reis C.P., Neufeld R.J., Ribeiro A.J., Veiga F. (2006). Nanoencapsulation I. Methods for preparation of drug-loaded polymeric nanoparticles, Nanomedicine Nanotechnology. Biol. Med..

[17] Pathak Y., Thassu D. (2016). Drug Delivery Nanoparticles Formulation and Characterization.

[18] Xu X., Ho W., Zhang X., Bertrand N., Farokhzad O. (2015). Cancer nanomedicine: From targeted delivery to combination therapy. Trends Mol. Med..

[19] Chang E.H., Harford J.B., Eaton M.A.W., Boisseau P.M., Dube A., Hayeshi R., Swai H., Lee D.S. (2015). Nanomedicine: Past, present and future—A global perspective, Biochem. Biophys. Res. Commun..

[20] Finch G., Havel H., Analoui M., Barton R.W., Diwan A.R., Hennessy M., Reddy V., Sadrieh N., Tamarkin L., Wolfgang M. (2014). Nanomedicine drug development: A scientific symposium entitled “Charting a roadmap to commercialization”. AAPS J..

[21] Venkatraman S. (2014). Has nanomedicine lived up to its promise?. Nanotechnology.

[22] Ehmann F., Sakai-Kato K., Duncan R., de la Ossa D.H.P., Pita R., Vidal J.-M., Kohli A., Tothfalusi L., Sanh A., Tinton S. (2013). Next-generation nanomedicines and nanosimilars: EU Regulators’ initiatives relating to the development and evaluation of nanomedicines. Nanomedicine (Lond.).

[23] Arachchige M.C.M., Reshetnyak Y.K., Andreev O.A. (2015). Advanced targeted nanomedicine. J. Biotechnol..

[24] Torchilin V. (2000). Drug Targeting. Eur. J. Pharm. Sci..

[25] Dube A., Lemmer Y., Hayeshi R., Balogun M., Labuschagne P., Swai H., Kalombo L. (2013). State of the art and future directions in nanomedicine for tuberculosis. Expert Opin. Drug Deliv..

[26] Kreuter J. (2014). Drug delivery to the central nervous system by polymeric nanoparticles: What do we know?. Adv. Drug Deliv. Rev..

[27] Tosi G., Vandelli M.A., Forni F., Ruozi B. (2015). Nanomedicine and neurodegenerative disorders: So close yet so far. Expert Opin. Drug Deliv..

[28] Venditto V.J., Szoka F.C. (2013). Cancer nanomedicines: So many papers and so few drugs!. Adv. Drug Deliv. Rev..

[29] Hare J.I., Lammers T., Ashford M.B., Puri S., Storm G., Barry S.T. (2016). Challenges and strategies in anti-cancer nanomedicine development: An industry perspective. Adv. Drug Deliv. Rev..

[30] Danhier F., Ansorena E., Silva J.M., Coco R., le Breton A., Préat V. (2012). PLGA-based nanoparticles: An overview of biomedical applications. J. Control. Release.

[31] Jiang W., von Roemeling C.A., Chen Y., Qie Y., Liu X., Chen J., Kim B.Y.S. (2017). Designing nanomedicine for immuno-oncology. Nat. Biomed. Eng..

[32] Mitragotri S., Anderson D.G., Chen X., Chow E.K., Ho D., Kabanov A.V., Karp J.M., Kataoka K., Mirkin C.A., Petrosko S.H. (2015). Accelerating the Translation of Nanomaterials in Biomedicine. ACS Nano.

[33] Dawidczyk C.M., Kim C., Park J.H., Russell L.M., Lee K.H., Pomper M.G., Searson P.C. (2014). State-of-the-Art in Design Rules for Drug Delivery Platforms: Lessons from FDA-approved Nanomedicines. J. Control. Release.

[34] Bawa R., Audette G.F., Reese B.E. (2016). Clinical Nanomedicine.

[35] Fischer S. (2014). Regulating nanomedicine. IEEE Pulse.

[36] KHoward A., Vorup-jensen T., Peer D. (2016). Nanomedicine.

[37] Liu D., Yang F., Xiong F., Gu N. (2016). The smart drug delivery system and its clinical potential. Theranostics.

[38] Wicki A., Witzigmann D., Balasubramanian V., Huwyler J. (2015). Nanomedicine in cancer therapy: Challenges, opportunities, and clinical applications. J. Control. Release.

[39] Smith J.A., Mathew L., Burney M., Nyshadham P., Coleman R.L. (2016). Equivalency challenge: Evaluation of Lipodox? as the generic equivalent for Doxil? in a human ovarian cancer orthotropic mouse model. Gynecol. Oncol..

[40] Moghimi M., Farhangrazi S. (2014). Defining and characterizing non-biological complex drugs (NBCDs)—Is size enough? The case for liposomal doxorubicin generics (“liposomal nanosimilars”) for injection. GaBI J..

[41] Bölükbas D.A., Meiners S. (2015). Lung cancer nanomedicine: Potentials and pitfalls. Nanomedicine (Lond.).

[42] Kemp J.A., Shim M.S., Heo C.Y., Kwon Y.J. (2016). “Combo” nanomedicine: Co-delivery of multi-modal therapeutics for efficient, targeted, and safe cancer therapy. Adv. Drug Deliv. Rev..

[43] Anselmo A.C., Mitragotri S. (2016). Nanoparticles in the Clinic. Bioeng. Transl. Med..

[44] Svenson S. (2014). What nanomedicine in the clinic right now really forms nanoparticles?. Wiley Interdiscip. Rev. Nanomed. Nanobiotechnol..

[45] Lammers T., Rizzo L.Y., Storm G., Kiessling F. (2012). Personalized nanomedicine. Clin. Cancer Res..

[46] Veiseh O., Tang B.C., Whitehead K.A., Anderson D.G., Langer R. (2015). Managing diabetes with nanomedicine: Challenges and opportunities. Nat. Rev. Drug Discov..

[47] (2017). National Center for Advancing Translational Sciences, Genetic and Rare Diseases Information Center. https://ncats.nih.gov/.

[48] Parveen S., Sahoo S.K. (2006). Nanomedicine: Clinical applications of polyethylene glycol conjugated proteins and drugs. Clin. Pharmacokinet..

[49] Vamvakas S., Martinalbo J., Pita R., Isaac M. (2011). On the edge of new technologies (advanced therapies, nanomedicines). Drug Discov. Today Technol..

[50] Stark W.J., Stoessel P.R., Wohlleben W., Hafner A. (2015). Industrial applications of nanoparticles. Chem. Soc. Rev..

[51] Hafner A., Lovrić J., Lakoš G.P., Pepić I. (2014). Nanotherapeutics in the EU: An overview on current state and future directions. Int. J. Nanomed..

[52] Woodson T.S. (2015). Public private partnerships and emerging technologies: A look at nanomedicine for diseases of poverty. Res. Policy.

[53] Islan G.A., Durán M., Cacicedo M.L., Nakazato G., Kobayashi R.K.T., Martinez D.S.T., Castro G.R., Durán N. (2017). Nanopharmaceuticals as a solution to neglected diseases: Is it possible?. Acta Trop..

[54] Bosetti R. (2015). Cost-effectiveness of nanomedicine: The path to a future successful and dominant market?. Nanomedicine.

[55] Béduneau A., Hindré F., Clavreul A., Leroux J.C., Saulnier P., Benoit J.P. (2008). Brain targeting using novel lipid nanovectors. J. Control. Release.

[56] Mato-Berciano A., Raimondi G., Maliandi M.V., Alemany R., Montoliu L., Fillat C. (2017). A NOTCH-sensitive uPAR-regulated oncolytic adenovirus effectively suppresses pancreatic tumor growth and triggers synergistic anticancer effects with gemcitabine and nab-paclitaxel. Oncotarget.

[57] Straussman R., Morikawa T., Shee K., Barzily-Rokni M., Qian Z.R., Du J., Davis A., Mongare M.M., Gould J., Frederick D.T. (2012). Tumour micro-environment elicits innate resistance to RAF inhibitors through HGF secretion. Nature.

[58] Stegh A.H. (2013). Toward personalized cancer nanomedicine—past, present, and future. Integr. Biol..

[59] Von Hoff D.D., Mita M.M., Ramanathan R.K., Weiss G.J., Mita A.C., Lorusso P.M., Burris H.A., Hart L.L., Low S.C., Parsons D.M. (2016). Phase I study of PSMA-targeted docetaxel-containing nanoparticle BIND-014 in patients with advanced solid tumors. Clin. Cancer Res..

[60] Lesniak A., Salvati A., Santos-Martinez M.J., Radomski M.W., Dawson K.A., Åberg C. (2013). Nanoparticle adhesion to the cell membrane and its effect on nanoparticle uptake efficiency. J. Am. Chem. Soc..

[61] Lynch I., Dawson K.A. (2008). Protein-nanoparticle interactions. Nano Today.

[62] Vauthier C., Ponchel G. (2016). Polymeric Nanoparticles on Nanomedicines: A Guide for Their Design, Preparation and Development.

[63] Nel A.E., Mädler L., Velegol D., Xia T., Hoek E.M.V., Somasundaran P., Klaessig F., Castranova V., Thompson M. (2009). Understanding biophysicochemical interactions at the nano-bio interface. Nat. Mater..

[64] Desai N., Trieu V., Damascelli B., Soon-Shiong P. (2009). SPARC Expression Correlates with Tumor Response to Albumin-Bound Paclitaxel in Head and Neck Cancer Patients. Transl. Oncol..

[65] Liu X., Kaminski M.D., Chen H., Torno M., Taylor L., Rosengart A.J. (2007). Synthesis and characterization of highly-magnetic biodegradable poly(d,l-lactide-co-glycolide) nanospheres. J. Control. Release.

[66] Okassa L.N., Marchais H., Douziech-Eyrolles L., Hervé K., Cohen-Jonathan S., Munnier E., Soucé M., Linassier C., Dubois P., Chourpa I. (2007). Optimization of iron oxide nanoparticles encapsulation within poly(d,l-lactide-co-glycolide) sub-micron particles. Eur. J. Pharm. Biopharm..

[67] Nkansah M.K., Thakral D., Shapiro E.M. (2011). Magnetic poly(lactide-co-glycolide) and cellulose particles for MRI-based cell tracking. Magn. Reson. Med..

[68] Sun Y., Zheng Y., Ran H., Zhou Y., Shen H., Chen Y., Chen H., Krupka T.M., Li A., Li P. (2012). Superparamagnetic PLGA-iron oxide microcapsules for dual-modality US/MR imaging and high intensity focused US breast cancer ablation. Biomaterials.

[69] Gatenby R.A., Gillies R.J. (2007). A microenvironmental model of carcinogenesis. Nat. Rev. Cancer.

[70] Lamonte G., Tang X., Chen J.L., Wu J., Ding C.C., Keenan M.M., Sangokoya C., Kung H., Ilkayeva O., Boros L.G. (2013). Acidosis induces reprogramming of cellular metabolism to mitigate oxidative stress. Cancer Metab..

[71] Mahoney B.P., Raghunand N., Baggett B., Gillies R.J. (2003). Tumor acidity, ion trapping and chemotherapeutics. Biochem. Pharmacol..

[72] Wojtkowiak J.W., Verduzco D., Schramm K.J., Gillies R.J. (2011). Drug resistance and cellular adaptation to tumor acidic pH microenvironment. Mol. Pharm..

[73] Karanth H., Murthy R.S.R. (2007). pH-Sensitive liposomes—Principle and application in cancer therapy. J. Pharm. Pharmacol..

[74] Vormehr M., Schrörs B., Boegel S., Löwer M., Türeci Ö., Sahin U. (2015). Mutanome Engineered RNA Immunotherapy: Towards Patient-Centered Tumor Vaccination. J. Immunol. Res..

[75] Kloke B.-P., Britten C.M., Loquai C., Löwer M., Attig S., Bukur V., Bidmon N., Derhovanessian E., Diekmann J., Diken M. Abstract CT202: IVAC MUTANOME: Individualized vaccines for the treatment of cancer. Proceedings of the AACR 106th Annunal Meeting.

[76] Michielin O., Coukos G. (2015). Immuno-Oncology.

[77] Würmseher M., Firmin L. (2017). Nanobiotech in big pharma: A business perspective. Nanomedicine (Lond.).

[78] Bergamaschi E., Murphy F., Poland C.A., Mullins M., Costa A.L., Mcalea E., Tran L., Tofail S.A.M. (2015). Impact and effectiveness of risk mitigation strategies on the insurability of nanomaterial production: Evidences from industrial case studies, Wiley Interdiscip. Rev. Nanomed. Nanobiotechnol..

[79] Monteiro-Riviere N.A., Tran C.L. (2006). Nanotoxicology: Progress toward Nanomedicine.

[80] Adamski J., Godman B., Ofierska-Sujkowska G., Osińska B., Herholz H., Wendykowska K., Laius O., Jan S., Sermet C., Zara C. (2010). Risk sharing arrangements for pharmaceuticals: Potential considerations and recommendations for European payers. BMC Health Serv. Res..

[81] Kar S.K., Rath B. (2016). Real-World Data Analytics in Global Pharmaceutical Marketing. IUP J. Knowl. Manag..

[82] Nanotechnology Characterization Laboratory (2017). Assay Cascade Protocols.

[83] Nasr M. (2006). Implementation of Quality by Design (QbD): Status, Challenges and Next Steps.

